# On Making Statistical Inferences Regarding the Relationship between Spawners and Recruits and the Irresolute Case of Western Atlantic Bluefin Tuna (*Thunnus thynnus*)

**DOI:** 10.1371/journal.pone.0156767

**Published:** 2016-06-07

**Authors:** Clay E. Porch, Matthew V. Lauretta

**Affiliations:** Southeast Fisheries Science Center, National Marine Fisheries Service, Miami, Florida, United States of America; Technical University of Denmark, DENMARK

## Abstract

Forecasts of the future abundance of western Atlantic bluefin tuna (*Thunnus thynnus*) have, for nearly two decades, been based on two competing views of future recruitment potential: (1) a “low” recruitment scenario based on hockey-stick (two-line) curve where the expected level of recruitment is set equal to the geometric mean of the recruitment estimates for the years after a supposed regime-shift in 1975, and (2) a “high” recruitment scenario based on a Beverton-Holt curve fit to the time series of spawner-recruit pairs beginning in 1970. Several investigators inferred the relative plausibility of these two scenarios based on measures of their ability to fit estimates of spawning biomass and recruitment derived from stock assessment outputs. Typically, these comparisons have assumed the assessment estimates of spawning biomass are known without error. It is shown here that ignoring error in the spawning biomass estimates can predispose model-choice approaches to favor the regime-shift hypothesis over the Beverton-Holt curve with higher recruitment potential. When the variance of the observation error approaches that which is typically estimated for assessment outputs, the same model-choice approaches tend to favor the single Beverton-Holt curve. For this and other reasons, it is argued that standard model-choice approaches are insufficient to make the case for a regime shift in the recruitment dynamics of western Atlantic bluefin tuna. A more fruitful course of action may be to move away from the current high/low recruitment dichotomy and focus instead on adopting biological reference points and management procedures that are robust to these and other sources of uncertainty.

## Introduction

The relationship between spawning capacity and the number of young fish that subsequently recruit to the population is an important factor in determining the productivity of fish stocks and the level of harvest they can sustain. Ideally, the functional form of the spawner-recruit (S-R) relationship would be deduced from process studies and its parameters estimated by fitting the function to a time series of independent measures of spawning capacity (e.g., egg production) and corresponding recruitment. More often than not, however, the functional form is unknown and independent measures of spawning capacity and recruits are unavailable. Consequently, it has become common practice to infer the nature of the S-R relationship by comparing the fits of a few candidate S-R models to estimates of spawners and recruits that are themselves derived from a model, such as the output from a stock assessment. This practice has contributed to a long-standing debate about the extent to which recruitment is related to spawning capacity.

Myers and Barrowman [1] concluded that there was a clear statistical relationship between spawners and recruits based on statistical inferences conducted on several hundred data sets (mostly stock assessment outputs). Gilbert [2] and others have argued that this apparent statistical relationship was often spurious and that low recruitment tended to drive declines in spawners rather than the reverse. More recently, Vert-pre et al. [3] used model selection criteria to infer that, for most of the stocks they examined from the RAM Legacy Stock Assessment Data Base, changes in surplus production were better explained by temporal shifts in the mean (as might occur with a change in the environment) than by trends in population abundance. Szuwalski et al. [4]) similarly used Spearman’s rank correlations to infer that the environment more strongly influences recruitment than spawning biomass over the observed stock sizes for many stocks. However, simulations conducted by both studies were seldom able to correctly identify a simulated stock driven by spawning biomass unless the variance in recruitment and “steepness” of the underlying S-R relationship were both low [4–5].

The debate over the cause of recruitment fluctuations is manifest in the provision of management advice for western Atlantic bluefin tuna (*Thunnus thynnus*) to the International Commission for the Conservation of Atlantic Tunas (ICCAT). For nearly two decades, the ICCAT Standing Committee for Research and Statistics (SCRS) has based forecasts of future abundance on two views of future recruitment potential: (1) a “low” recruitment scenario based on hockey-stick (two-line) curve where the expected level of recruitment is set equal to the geometric mean of the recruitment estimates for the years after 1975, and (2) a “high” recruitment scenario based on a Beverton-Holt curve fit to the time series of spawner-recruit estimates beginning in 1970. The original version of the “low” recruitment potential scenario was adopted in the early 1990s simply to provide reasonable values for short-term forecasts of spawning biomass under various catch levels and later as an alternative to the “base” Beverton-Holt model (p. 200 in [6]). However, during the late 1990s, arguments began to be advanced that the low recruitment scenario represented a permanent state of nature and that the ICCAT objective of maximum sustainable yield should be based on that assumption [7]. Proponents of this view rationalized that the high recruitments estimated during the 1960s and early 1970s reflected a relatively favorable environmental regime and the drop in 1976 to relatively low levels reflected a rapid shift to a less favorable regime that prevails to this day. Others pointed out that recruitment trends alone are insufficient to substantiate this hypothesis [8] and suggested that the decrease in recruitment is better correlated with spawning biomass than it was with any known environmental cue [9–10].

The possibility of an environmental explanation for the change in recruitment was formally acknowledged by the SCRS in 2002 (p. 76 in [11]) and the SCRS has been irresolute on the matter ever since, choosing to include the implications of both the high and low recruitment scenarios in its management advice with the caveat that “The Committee has no strong evidence to favor either scenario over the other and notes that both are reasonable (but not extreme) lower and upper bounds on rebuilding potential” (see, e.g., [12]). Several attempts have been made to break this deadlock using statistical inference. McAllister et al. [8] computed Bayes posterior probabilities for the fits of the “high” and “low” recruitment models to estimates of spawners and recruits from the 1998 stock assessment and suggested that the empirical weight for the low recruitment scenario was so low as to warrant its exclusion from further consideration. Rosenberg et al. [13] applied similar inferential techniques (based on F-tests and Bayes factors) to spawner-recruit estimates from the 2010 assessment. They also found somewhat more support for the “high” recruitment scenario than for the two-line model, but concluded that the use of any particular S-R relationship had little foundation in the spawner and recruitment estimates from the assessment model. More recently, a method was proposed at the 2014 SCRS West Atlantic Bluefin Tuna Species Group Meeting that used the bias-corrected Akaike Information Criterion (AICc) as a measure of each model’s ability to fit to spawner and recruitment estimates derived from the 2012 and 2014 stock assessments [14]. The results of this analysis indicated that the AICc weights strongly favored a three-line model which incorporated regime shift after 1975 over the Beverton-Holt model. As a result, the SCRS agreed to incorporate a subtle, but important addition to the usual caveat in its management advice (p. 110 in [15]) that reads “A preliminary analysis conducted after the assessment meeting indicated an improved fit of assessment outputs by the low recruitment potential hypothesis; however, the Committee could not agree whether this provided sufficient evidence to favour that scenario, in light of prior analyses that gave conflicting conclusions.”

Some members of the SCRS expressed concern that the approach used by the bluefin working group [14] was based on estimates derived from the stock assessment model that may be subject to a number of biases. Moreover, the likelihood expressions assumed that spawning biomass is known without error, when in fact the errors in the stock assessment estimates of spawning biomass are comparable to, or may even exceed, the errors in recruitment. Failing to account for large observation errors in the estimates of spawning biomass will lead to statistically inconsistent estimates [16,17], which in turn could bias the interpretations of goodness-of-fit criteria such as AICc. This paper proposes an errors-in-variables solution to the problem and demonstrates that the failure to accommodate errors in the estimates of spawning biomass can predispose statistical inferences in favor of regime-shift hypotheses.

## Material and Methods

The methods used by the SCRS bluefin working group [14] and several similar efforts assume that spawning biomass (*s*) values are known without error and that the “observed” recruitments *R* are subject to a multiplicative error such that

$$\;\;R_{y}=r\left(s_{y-1}|\theta \right)e^{\delta_{y}}$$ (1)

where *y* indexes the year, *r*(*s|θ*) represents the candidate S-R relationship, *θ* represents a vector of *k* parameters and the *δ*_y_ represent independent, normally-distributed, random variables with mean 0 and variance σ^2^. Maximum likelihood estimates of the parameters *θ* and σ^2^ can then be obtained from the time series of *R* and *s* by minimizing the negative log-likelihood function corresponding to eq (1):

$$-LL=0.5\left(n\text{ln}\left(\sigma_{}^{2}\right)+\overset{Y}{\underset{y=1971}{\sum }}{\left(\frac{\text{ln}\left(\frac{R_{y}}{r\left(s_{y-1}|\theta \right)}\right)}{\sigma }\right)}^{2}{}^{}\right)$$ (2)

where *n* is the number of year-classes in the sample and Y is the last year used in the analysis.

In the case of western Atlantic bluefin tuna, however, the estimates of spawning biomass and recruitment from the stock assessment are uncertain. Accordingly, a more reasonable statistical model is

$$R_{y}=r\left(s_{y-1}|\theta \right)e^{\delta_{y}+\epsilon_{y}}$$ (3)

$$S_{y}=s_{y-1}e^{\varepsilon_{y}}$$

Here the *δ*_y_ represent process errors and are treated as independent random normal variables with mean zero and variance *σ*^2^ as in the case of model (1). The terms *∈*_*y*_ and *ε*_*y*_ represent the “observation” errors in recruits and spawning biomass, respectively, which we also treat as random normal variables, although they do not necessarily need to be independent. The method of Ludwig and Walters [16], for example, is a special case of eq (3) where *∈*_*y*_ = *ε*_*y*_. They reasoned that, for the salmon populations they studied, recruits were usually estimated as catch plus spawners and catch was known with little error; therefore, the observation error for the number of recruits and spawning biomass should be similar for a given generation. Bluefin tuna, on the other hand, have a long generation time and the catches are not without error, therefore the estimates of recruitment and spawning biomass in the same year are not likely to be closely correlated, and we have preferred instead to make the less restrictive assumption that observation errors *∈*_*y*_ and *ε*_*y*_ are statistically independent, random normal variables with similar variance $\sigma_{\epsilon }^{2}$. Kendall and Stuart [18] have shown that it is not possible to estimate both the process variance and observation variance without additional information, therefore we follow [16] in prescribing the ratio

$$\sigma_{\epsilon }^{2}=\tau \sigma^{2}$$ (4)

where *τ* represents a scalar between the observation and process errors.

Under these conditions, the probability density of observing the pair of observations [*R*_*y*_,*S*_*y-1*_] is bivariate normal and the parameters *θ* and *s*_y_ may all be estimated by minimizing the negative log-likelihood function

$$-LL=0.5\left(n\text{ln}\left(\sigma_{R}^{2}\right)+n\text{ln}\left(\sigma_{S}^{2}\right)+\overset{Y}{\underset{y=1971}{\sum }}{\left(\frac{\text{ln}\left(\frac{R_{y}}{r\left(s_{y-1}|\theta \right)}\right)}{\sigma_{R}^{}}\right)}^{2}+{\left(\frac{\text{ln}\left(S_{y-1}/s_{y-1})\right)}{\sigma_{S}^{}}\right)}^{2}{}^{}\right)$$ (5)

where $\sigma_{R}^{2}=\left(1+\tau \right)\sigma^{2}$ and $\sigma_{S}^{2}=\tau \sigma^{2}$.

An approximately bias-corrected estimate of *σ*^2^ can be obtained from the residual sum of squares as

$$\;{\hat{\sigma }}^{2}=\frac{1}{n-k}\sum_{y=1971}^{Y}\left[\frac{{\left(\text{ln}\left(R_{y}/r(s_{y-1}|\theta )\right)\right)}^{2}}{1+\tau }+\frac{{\left(\text{ln}\left(S_{y-1}/s_{y-1}\right)\right)}^{2}}{\tau }\right]$$ (6)

where the denominator reflects the number of data points (2*n*) less the number of estimated parameters besides *σ* itself (*n*+*k*).

The two alternative S-R models examined by the bluefin working group [14] included the Beverton-Holt model (eq 7, the high recruitment potential scenario) and a “three-line” model intended to reflect a hypothesized regime shift to a less productive state (eq 8, the low recruitment potential scenario):

$$r\left(s_{y-1}|\alpha ,\beta \right)=\frac{\alpha s_{y-1}}{\beta +s_{y-1}}$$ (7)

$$r\left(s_{y-1}|\mu_{1},\mu_{2},\gamma ,\omega \right)=\{\begin{array}{ll} \mu_{1} & \text{if}\;y\leq \;\omega \\ \mu_{2} & \text{if}\;y>\;\omega ,\;\;s_{y-1}\geq \;\gamma \\ \mu_{2}s_{y-1}/\gamma & \text{if}\;y>\;\omega ,\;\;s_{y-1}<\;\gamma \end{array}$$ (8)

where ω is the last year of the first “regime” and γ is the spawning biomass threshold (inflection point) below which recruitment begins to decline.

The “three-line” model is, by design, relatively insensitive to observations errors in *S* because the predictor of recruitment is by definition independent of spawning biomass (d*R*/d*s* = 0) except at the transition from one “regime” to the other and the position of the inflection point. It is undefined when the observation error in *R* is negligible compared to *S* since d*S*/d*r* = ∞. Accordingly, it does not lend itself well to hypothesis testing when there are errors in both *R* and *S*. However, as pointed out by Rosenberg et al. [13], it may be more appropriate to use the same functional model for both regimes under the supposition that the underlying processes controlling recruitment are similar, but occur at different scales. One alternative is to assume a Beverton-Holt relationship applies during both regimes, but with different parameters:

$$r\left(s_{y-1}|\alpha_{1},\beta_{1},\alpha_{2},\beta_{2},\omega \right)=\{\begin{array}{ll} \frac{\alpha_{1}s_{y-1}}{\beta_{1}+s_{y-1}} & \text{if}\;y\leq \;\omega \\ \frac{\alpha_{2}s_{y-1}}{\beta_{2}+s_{y-1}} & \text{if}\;y>\;\omega \end{array}$$ (9)

For convenience, the parameters *α* and *β* for the Beverton-Holt function have been translated into more intuitive metrics; the recruitment at the unfished level (*r*_0_) and steepness (*h*, the proportion of *r*_0_ produced at 20% of the unfished level of spawning biomass):

$$r_{0}=\alpha -\frac{\beta }{\varphi_{0}}$$ (10)

$$h=\frac{\beta +r_{0}\varphi_{0}}{5\beta +r_{0}\varphi_{0}}$$ (11)

where *φ*_0_ is the estimated tonnage of unfished spawning biomass per recruit obtained from the assessment (0.7 t).

Maximum likelihood estimates were obtained for the parameters of models 7–9 via minimization of the negative log-likelihood expression (eqs 5 and 6) under various levels of the ratio of observation to process variance (τ). Minimizations were accomplished using GRG nonlinear routine with automatic scaling in Microsoft Excel Solver. The spawning stock biomass and recruitment estimates used in the analysis (Table 1) were taken from the 2014 assessment of western Atlantic bluefin tuna [19].

**Table 1:** Spawning stock biomass (t) and recruitment estimates (in number) from the 2014 stock assessment (note that results for the last three years were not included in the AICc computations because they were considered to be poorly determined by the assessment working group).

Year	SSB	Recruitment (Age 1)
1970	51113	363640
1971	50857	322392
1972	51266	278521
1973	51539	150973
1974	46241	465746
1975	41025	164391
1976	36159	135241
1977	31021	112512
1978	27718	95145
1979	24534	99656
1980	22252	81299
1981	19138	80599
1982	18020	82285
1983	17279	104287
1984	16438	93252
1985	14850	98867
1986	15239	102505
1987	14630	91424
1988	14523	138821
1989	14103	121629
1990	13546	114105
1991	13283	94800
1992	12927	83580
1993	13133	77333
1994	13055	88548
1995	13721	114612
1996	14996	92054
1997	16121	75317
1998	16494	101446
1999	16136	104719
2000	16445	90853
2001	16249	91803
2002	16103	105420
2003	16178	173337
2004	16797	149469
2005	17324	63186
2006	18047	86729
2007	20301	96287
2008	21323	74561
2009	21706	65547
2010	22700	80317

The AICc was calculated for each model

$$AICc=2\cdot k\left(\frac{n}{n-k-1}\right)-2\cdot LL$$ (12)

The relative probability that the *i*th of a collection of models provides the most parsimonious explanation of the data (minimizes the estimated ‘information loss’) was computed as

$$P_{i}=\frac{e^{\Delta_{i}/2}}{\sum_{i}e^{\Delta_{i}/2}}$$ (13)

where Δ_i_ is the difference between the lowest AICc value among all candidate models and the AICc of model *i* [20]. In the present case the relevant comparisons are between models 7 and 8 or models 7 and 9.

## Results

**Fig 1:**
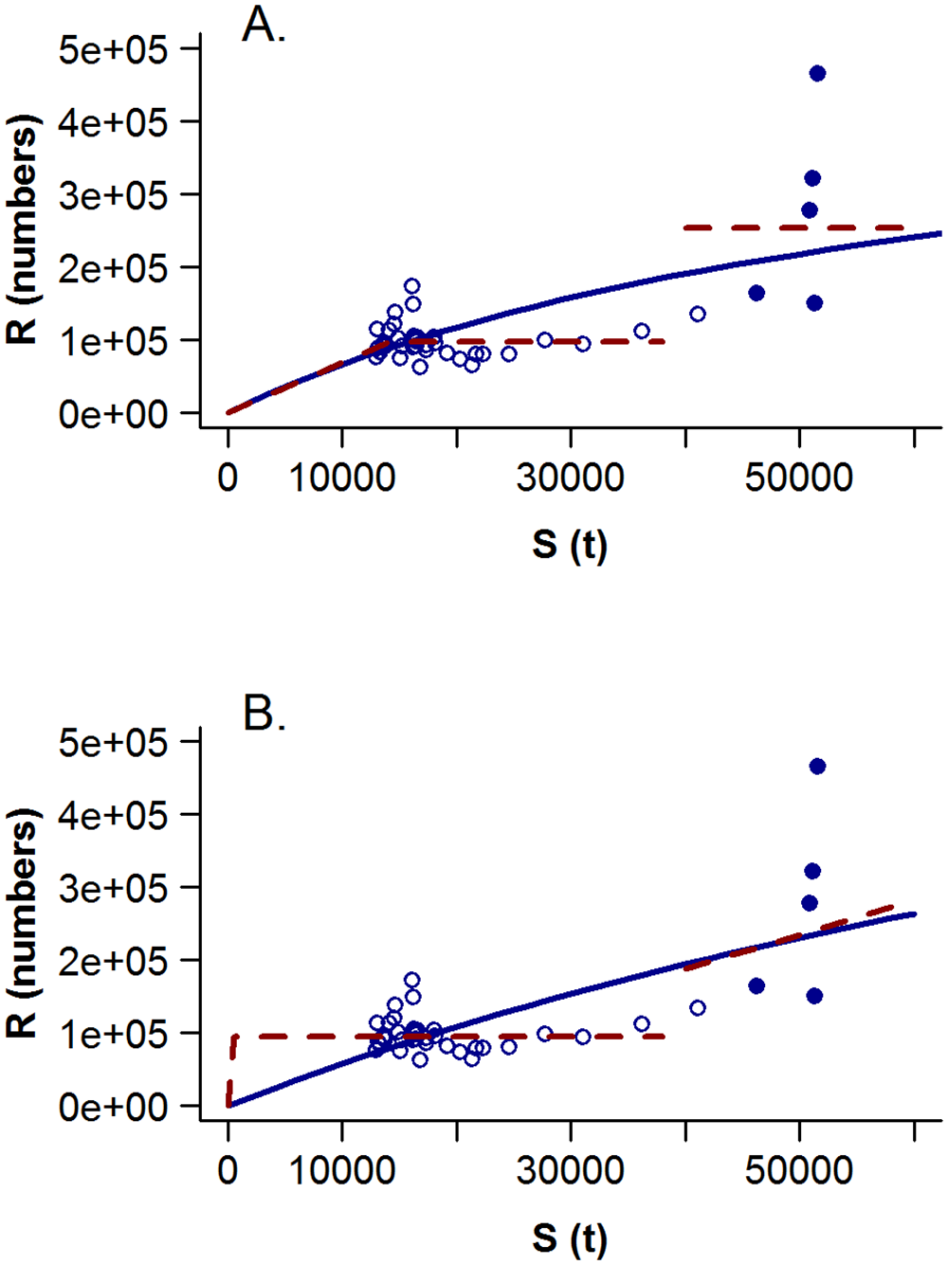
Candidate spawner-recruitment relationships fit to stock assessment outputs for western Atlantic bluefin tuna assuming τ = 1. The solid circles represent the year-classes from 1970 to 1975 and the hollow circles represent subsequent year-classes. The solid lines represent the Beverton-Holt curve fit to the entire time series and the dashed lines represent A) the three-line regime-shift model and B) the Beverton-Holt regime shift model.

The maximum likelihood estimates of the parameters for models 7–9 are tabulated for a range of values of τ in Table 2 and the corresponding curves for τ = 1 are plotted in Fig 1. The estimates of the parameters of the Beverton-Holt model (7) are sensitive to the level of observation error assumed, with estimates of *h* decreasing and estimates of *r*_0_ increasing as the ratio *τ* increases (and therefore also the level of observation error *σ*_*∈*_). As the value of *τ* (and *σ*_*∈*_) decreases to zero, the parameter estimates converge to those obtained with no observation error (model 7: *h* = 0.59, *r*_0_ = 283844). As expected, the estimates of the parameters for the three-line model (8) are insensitive to the level of *τ* (and *σ*_*∈*_) because it cannot admit the possibility of a finite d*S*/d*r*. The estimates of *μ*_1_ and *ω* were identical for all values of *τ* and the values of *μ*_2_ and *γ* changed only slightly as τ varied. Interestingly, the parameters of the biphasic Beverton-Holt regime-shift model (9) were also rather insensitive to *τ* (and *σ*_*∈*_); the primary difference being a shift in *ω* from 1976 to 1977 for *τ* ≥0.5 with a corresponding minor change in values of the other parameters. The values of recruitment predicted for the three-line and biphasic Beverton-Holt regime-shift models were similar, except that the biphasic Beverton-Holt model admits some curvature for the high recruitments in the early years (Fig 1).

**Table 2:** Parameter estimates for the Beverton-Holt, three-line regime shift and Beverton-Holt regime shift models fitted to the spawning stock biomass and recruitment estimates from the 2014 assessment of western Atlantic bluefin tuna.

Beverton-Holt (eq 7)								
τ	α	β	*h*	*r*_0_	σ	σ_ε_	σ_R_	
10	3150170	563401	0.49	2343201	0.08	0.25	0.27	
5	3140258	561615	0.49	2335847	0.11	0.25	0.27	
2	1531554	262591	0.50	1155440	0.16	0.23	0.28	
1.5	1225748	205790	0.51	930991	0.18	0.22	0.29	
1.2	917571	148477	0.51	843203	0.20	0.22	0.29	
1	914441	147966	0.52	704904	0.21	0.21	0.30	
0.8	787806	124481	0.52	702507	0.22	0.20	0.30	
0.5	609582	91409	0.52	609510	0.25	0.18	0.31	
0.1	392281	51238	0.53	566689	0.31	0.10	0.33	
0.01	349168	43303	0.58	287144	0.33	0.03	0.33	
0	344690	42479	0.59	283844	0.33	na	0.33	
Three-line regime shift (eq 8)								
τ	μ_1_	μ_2_	ω	γ	σ	σ_ε_	σ_R_	
10	253066	97849	1976	14317	0.08	0.25	0.26	
5	253066	97807	1976	14282	0.11	0.24	0.26	
2	253066	97674	1976	14131	0.15	0.21	0.26	
1.5	253066	97695	1976	14188	0.17	0.20	0.26	
1.2	253066	97650	1976	14167	0.18	0.19	0.26	
1	253066	97600	1976	14103	0.18	0.18	0.26	
0.8	253066	97599	1976	14124	0.19	0.17	0.26	
0.5	253066	97650	1976	14176	0.21	0.15	0.26	
0.1	253066	97602	1976	14142	0.25	0.08	0.26	
0.01	253066	97559	1976	14109	0.26	0.03	0.26	
0	253066	97549	1976	14103	0.26	na	0.26	
Beverton-Holt regime shift (eq 9)								
τ	*h*_*1*_	*r*_0,1_	*h*_*2*_	*r*_0,2_	ω	σ	σ_ε_	σ_R_
10	0.45	8.E+09	1.00	95590	1977	0.07	0.23	0.24
5	0.45	8.E+09	1.00	95590	1977	0.10	0.22	0.24
2	0.45	8.E+09	1.00	95590	1977	0.14	0.20	0.24
1.5	0.45	8.E+09	1.00	95590	1977	0.16	0.19	0.25
1.2	0.45	8.E+09	1.00	95590	1977	0.17	0.18	0.25
1	0.45	8.E+09	1.00	95590	1977	0.18	0.18	0.25
0.8	0.45	8.E+09	1.00	95590	1977	0.19	0.17	0.25
0.5	0.45	8.E+09	1.00	95590	1977	0.21	0.15	0.25
0.1	0.47	2.E+09	1.00	96542	1976	0.25	0.08	0.26
0.01	0.47	2.E+09	1.00	96542	1976	0.26	0.03	0.26
0	0.47	2.E+09	1.00	96548	1976	0.26	na	0.26

**Fig 2:**
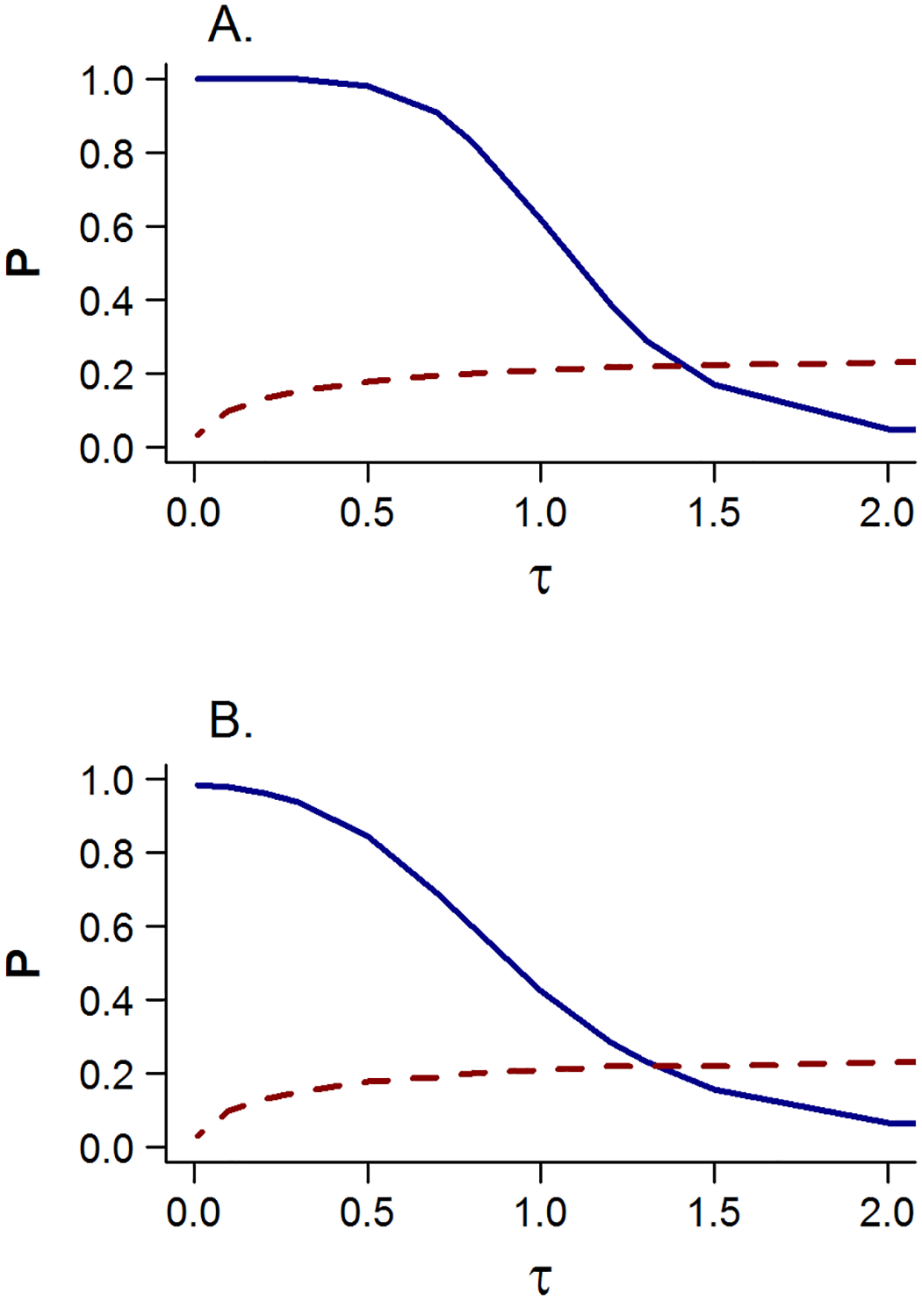
Relative AICc statistical weights *P* assigned to the regime shift hypothesis (solid line) and the estimated standard deviation of the observation error (dashed line). The upper plot (A) represents the three-line regime shift model and the lower plot (B) represents the bi-phasic Beverton-Holt (regime shift) model. Note that the weights in this case are computed for the two separate pairs of models, i.e., eq 13 is applied to (A) the single Beverton-Holt and three-line models and (B) the single and bi-phasic Beverton-Holt models.

The negative log-likelihoods (-LL) and AICc statistics corresponding to each model are summarized for a range of *τ* in Table 3 and Fig 2. The biphasic Beverton-Holt model provided the best fit to the data (lowest negative log-likelihoods) for all values of *τ*. However, the model providing the most parsimonious fit according to the AICc criteria *P* depended strongly on *τ* The single-phase Beverton-Holt model provided the most parsimonious fit for *τ*>1.2 and the three-line model was the most parsimonious otherwise.

**Table 3:** Comparison of negative log-likelihoods and AICc between the Beverton-Holt, three-line regime shift and Beverton-Holt regime shift models fitted to the spawning stock biomass and recruitment estimates from the 2014 assessment of western Atlantic bluefin tuna. Note that the AIC weights *P* were computed here by applying eq 13 to all three models.

	Beverton-Holt (no regime shift)			Three-line regime shift			Beverton-Holt regime shift		
τ	-LL	AICc	*P*	-LL	AICc	*P*	-LL	AICc	*P*
10	-89.04	13.03	1.00	-91.40	28.96	0.00	-98.72	25.59	0.00
5	-89.24	12.63	0.99	-93.12	25.53	0.00	-100.05	22.93	0.01
2	-90.18	10.75	0.89	-97.51	16.74	0.04	-103.50	16.04	0.06
1.5	-90.86	9.38	0.72	-99.59	12.58	0.15	-105.14	12.74	0.13
1.2	-91.59	7.93	0.49	-101.47	8.83	0.31	-106.65	9.73	0.20
1	-92.38	6.35	0.30	-103.18	5.41	0.48	-108.04	6.95	0.22
0.8	-93.57	3.98	0.13	-105.50	0.77	0.66	-109.94	3.15	0.20
0.5	-97.04	-2.97	0.02	-111.17	-10.58	0.88	-114.68	-6.34	0.11
0.1	-118.52	-45.93	0.00	-136.92	-62.07	0.99	-138.25	-53.48	0.01
0.01	-161.55	-131.99	0.00	-181.15	-150.54	0.99	-181.60	-140.18	0.01
0	-25.01	-43.36	0.00	-35.42	-59.07	0.73	-35.84	-57.13	0.27

## Discussion

The results of this study are consistent with previous studies [16,17,21] in that they demonstrate the estimates for the parameters of the S-R relationship will tend to be biased when substantial observation errors in *S* are not accounted for. When the Beverton-Holt model is applied to bluefin tuna data, the bias will lead to the perception that the stock is more productive at low stock sizes (higher steepness). More importantly, the AICc weights used to compare alternative S-R relationships depend strongly on how the observation errors are modeled.

The analysis conducted by the SCRS Bluefin Tuna Working Group [14] assumed that there was no observation error in the estimates of spawning biomass obtained from the stock assessment and fixed the hinge point *γ* to a value specified based on visual inspection of the S-R plot (but did not count it as an estimated parameter as it should have been). In that special case, the AICc weights were overwhelmingly in favor of the three-line regime shift model. Accounting for the estimation of *γ* reduced the AICc weight given to the three-line model, but the three-line model was still favored (Table 3, *τ* = 0). Accommodating observation errors in *S* was much more influential, with the evidence shifting in favor of the single-phase Beverton-Holt model when the variance of the observation error exceeded that of the process error in *r* (Fig 2a). A very similar result was obtained when the three-line model was replaced by the more-flexible biphasic Beverton-Holt model (Fig 2b). To put this in perspective, it is important to compare the trends in the weights assigned to the regime shift hypothesis relative to the magnitude of the estimates of *σ*_*∈*_. The weights do not substantially favor the regime-shift hypotheses except where the value of *σ*_*∈*_ is less than 0.2 (i.e., the coefficient of variation of the observation error is less than 20%). For comparison, this level of *σ*_*∈*_ is similar to the bootstrap estimates of uncertainty derived from the 2014 assessment for the recent years of spawning biomass, which are likely underestimates because they do not account for uncertainties about stock mixing, natural mortality and aging the catch [12, 19]. When such uncertainties have been modeled, the standard errors are very much greater than 0.2, as shown by stock assessment sensitivity analyses on natural mortality and the catch aging method [12, 19].

Other variations of model (3) could be explored. One could, for example, treat the values of *s* as random effects, thereby reducing the number of parameters and perhaps achieving a more statistically-consistent estimator [22], but at the expense of having to assume the nature of the distribution of *s*. Another alternative might be to compare alternative recruitment hypotheses within a statistical catch-at-age assessment framework rather than fitting to the assessment outputs. However, the degree to which one S-R model might be favored over another, whether outside or within the assessment model, depends on other specifications in the assessment model, such as natural mortality or fishery selectivity [23] and, in the case of Atlantic bluefin tuna, mixing between the eastern and western subpopulations [24–26]. Moreover, it has been shown for several species that the use of spawning biomass as a proxy for reproductive capacity tends to underestimate the per-capita reproductive potential of older females [27–29], which can lead to erroneous interpretations of the S-R relationship. For these reasons, model comparisons alone are unlikely to provide a definitive answer as to the true nature of the S-R relationship and the degree to which it is influenced by environmental changes.

Klaer et al. [30] suggest a more systematic approach is needed that would include environmental and ecological studies that provide independent evidence of a process that would support the hypothesized regime shift. Little along these lines has been done to support the regime shift hypothesis for bluefin tuna. While Fromentin et al. [31] found that the environmental conditions in the equatorial Atlantic were more favorable for bluefin tuna during the 1960s than subsequently, they did not find evidence that this change directly affected the nature of the S-R relationship. Instead, they proposed that Western Atlantic stock may be less productive now than it was during the 1950s and 1960s in part because a migratory contingent that used to concentrate on feeding grounds off Brazil was discovered and overfished during the early 1960s.

Data obtained from direct surveys of the spawning and juvenile populations would not be subject to many of the potential biases associated with stock assessment outputs and therefore would be better suited for the types of analyses discussed in this paper. However, even where such data are available, the very act of managing the resource will work against a resolution to the issue. As Boettiger et al. [32] pointed out, model-choice approaches can be misleading, particularly when most of the data come from around a stable steady state such that all the parametric models are approximately linear and approximately identical. In the case of western Atlantic bluefin tuna, the management regulations promulgated over the last 30 years have worked to the effect of maintaining a relatively stable biomass and, without further reductions in catch, it seems unlikely that the spawning biomass will increase to a level that would provide enough contrast to identify the most appropriate model [33] even in the absence of any environmentally-driven shifts. We believe a more fruitful course of action for western Atlantic bluefin tuna is to move away from the current high/low recruitment dichotomy and focus instead on adopting biological reference points and management procedures that are robust to this and other sources of uncertainty. A similar strategy might be appropriate for Pacific bluefin tuna (*Thunnus orientalis*), Southern bluefin tuna (*Thunnus maccoyii*) and other species that were depleted before monitoring began and where there is little interest in adaptive resource management.
